# A Novel Elastomer-Based Inclinometer for Ultrasensitive Bridge Rotation Measurement

**DOI:** 10.3390/s22072715

**Published:** 2022-04-01

**Authors:** De Zhou, Ningbo Wang, Chaofeng Fu, Chuanrui Guo, Yangping Zhao

**Affiliations:** 1 School of Civil Engineering, Central South University, Changsha 410075, China; 210026@csu.edu.cn (D.Z.); wangnb@csu.edu.cn (N.W.); fuchaofeng970715@outlook.com (C.F.); 2 Institute of Urban Smart Transportation & Safety Maintenance, College of Civil and Transportation Engineering, Shenzhen University, Shenzhen 518060, China; yangping@szu.edu.cn

**Keywords:** inclinometer, bridge rotation, elastomer, health monitoring, ultrasensitive measurement

## Abstract

Bridge deformation consists of cross-section rotation and deflection, which are crucial parameters for bridge capacity evaluation and damage detection. The maximum value of deflection usually happens at mid-span while for rotation it happens at two-ends. Therefore, compared with deflection, rotation is more convenient for in-situ measurement since the bridge pier can be the reference point. In this study, a high-precision inclinometer for bridge rotation measurement was conceptualized, designed, and validated. The proposed inclinometer converted the small rotation of bridge section into the deformation of an elastomer. Strain gauges were then utilized to measure the elastomer deformation and thus the bridge rotation can be obtained. The dimensions and modulus of the elastomer were designed and chosen based on the theoretical analysis. Characteristics of the inclinometer were calibrated in lab and in-situ experiments at an in-service bridge were conducted to validate its feasibility and robustness. Test results showed that the proposed inclinometer had excellent performance in resolution and accuracy, which indicate its great potential for future bridge health monitoring.

## 1. Introduction

Load effects such as strain, deflection, rotation, and acceleration are commonly used for bridge capacity evaluation and damage detection [1,2,3,4,5,6,7,8]. Deflection reflects the overall deformation of a bridge structure. Current methods for bridge deflection monitoring usually measure the distance between the bridge and a fixed reference point. Based on the location of the reference point, different sensors/equipment can be used: linear variable differential transformer (LVDT) [9], total station [10], machine vision [11], laser vibrometer [12,13], radar [14], and global navigation satellite system (GNSS) [15,16]. LVDT-based monitoring is a contact method with high accuracy for dynamic measurement. Machine vision, laser vibrometer, and other non-contact monitoring methods are characterized by relatively low precision and insufficient resolution, and thus are not suitable for dynamic or real-time monitoring. They can be used for monitoring large-scale or long-term deflection. Generally, the maximum deflection of a bridge structure occurs in the mid-span section, making it difficult to monitor since no fixed reference point is available. For this reason, deflection measurement is limited in practical application for bridge evaluation.

Similar to deflection, bridge rotation also reflects the overall deformation of a bridge structure. Compared with deflection, the rotation of a bridge section can be measured relative to the vertical direction without using any physical reference point. Therefore, rotation measurement for bridge health monitoring has gradually become a popular research topic. However, rotation measurement is challenging in conventional understanding since the rotation absolute value for any civil engineering infrastructure is limited and not on a magnified scale. To address this issue, many researchers developed various techniques for rotation measurement in the past decade [17,18,19]. So far, rotation monitoring methods are generally based on the gravity pendulum setup and then convert or magnify the angular information into other types of measurable parameters such as resistance, capacitance, magnetic field, or optical parameters [20], etc.

In many studies, the rotation data of different bridge sections have been used to indirectly calculate or reconstruct the deflection and deformation of the bridge superstructure [21,22,23,24]. Zhai et al. [25] proposed a method to identify the modal parameters of a simply supported beam based on the inclination angle data. Hoult [26] used a rotation sensor network to monitor the long-term changes in boundary conditions of long-span bridges. Many recent studies also have used rotation data for the damage detection [27,28]. Alamdari et al. [29] proposed a rotation-based method for identifying the stiffness loss of a cable stayed bridge, and compared experimental results with the strain-based damage identification method. The results showed that the effectiveness of the strain-based method was largely dependent on the position of the strain-measuring points, which may affect the cable damage identification results. Huseynov et al. [20] proposed a new method to identify the presence and location of damage in a simply supported bridge based on the characteristics of rotation influence lines and verified its effectiveness through laboratory experiments. The study concluded that rotation is the optimum parameter for identifying local damage.

Compared with local strain and acceleration measurement, rotation can better reflect the overall deformation of a structure. Moreover, rotation is also more convenient to measure than deflection since no extra reference point is needed. Thus, it has great application prospects and supplementary potential in the current bridge health monitoring and condition evaluation system [30,31]. Inclinometers have been widely used in automobile, aviation, and other industrial fields for rotation measurement. In civil engineering, inclinometers were originally used for geotechnical monitoring and long-term deformation monitoring of bridges during the construction or early service stage [32,33]. Moreover, inclinometers are also useful to assess the effective plastic deformation demand during seismic events [34,35]. In the past decades, with the improvement in precision of the inclinometer, rotation monitoring has been used increasingly in the civil engineering for structural health monitoring. Table 1 lists some commercial inclinometers and their parameters [20,36,37,38,39,40]. Among them, precision of 3.5 × 10^−4^ (°) and resolution of 1 × 10^−4^ (°) partially meet the minimum requirements for small-angle bridge monitoring. However, it is evident from Table 1 that the sampling frequencies of such inclinometers are too low to meet the demands of dynamic tests. 

In summary, current inclinometers have the following disadvantages for bridge rotation monitoring: (1) The working range is too wide while the resolution and precision are too low. Bridge rotation is very small in amplitude [41,42] and thus it is difficult to capture the tilt signal. (2) Current inclinometers are designed primarily for static or low-frequency monitoring, which limits their use in dynamic measurements.

To address above issues, the novelty of this work is to propose, design, and validate a new elastomer-based inclinometer with high accuracy for ultrasensitive dynamic monitoring of beam-end rotation. The motivation of this research is to monitor the health condition and evaluate the bridge bearing capacity in more accurate and efficient ways by utilizing the bridge rotation response. The working principle of the proposed inclinometer is to convert or magnify the beam-end rotation into the deformation of an elastomer. Based on the dynamic measurement system, a custom software module was developed for data acquisition. Characteristics of the proposed inclinometer, including range, resolution, linearity, and repeatability were calibrated first in the lab. Finally, to verify its feasibility and effectiveness, in-situ experiments were conducted to measure the dynamic rotation response of an in-service bridge subjected to moving vehicle loads. The in-situ test results indicate that the proposed inclinometer has excellent accuracy and resolution for bridge rotation measurement and thus has great potential for future bridge health monitoring and capacity evaluation.

This paper is organized into the following sections:

Section 1 is the general introduction of research background, current trend of rotation measurement, and the novelty and motivation of this work. Section 2 is dedicated to the working principle of the elastomer-based inclinometer. Section 3 constructs the design and data acquisition system of the proposed inclinometer. Section 4 investigates the characteristics of the proposed inclinometer, including range, resolution, linearity, and repeatability. Section 5 describes the in-situ experimental tests to illustrate the feasibility of the proposed method. Finally, discussions and conclusions are presented in Section 6.

## 2. Working Principle of the Elastomer-Based Inclinometer

**Figure 1:**
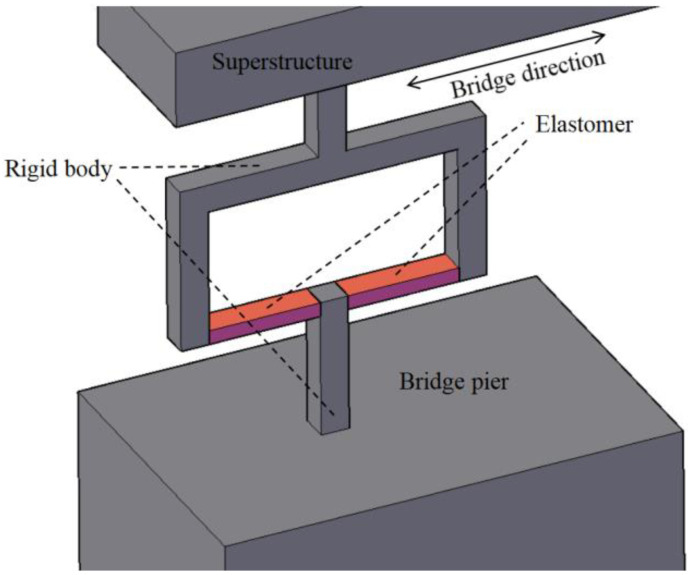
Schematic illustration of the inclinometer setup.

The fundamental principle of the proposed inclinometer is to convert the section rotation into the strain of an elastomer. By utilizing the strain gauges on elastomer, high-precision monitoring of the rotation can be achieved. In this method, bridge pier is used as a reference point, relative to which the beam-end rotation is monitored. The concept of this method is shown in Figure 1, top and bottom rigid bodies are fixed on the pier and superstructure, respectively. The elastomer (red part) is fixed to the two rigid bodies for connection and transfers the section rotation into elastomer bending. The bending strain can then be measured and utilized for rotation sensing.

**Figure 2:**
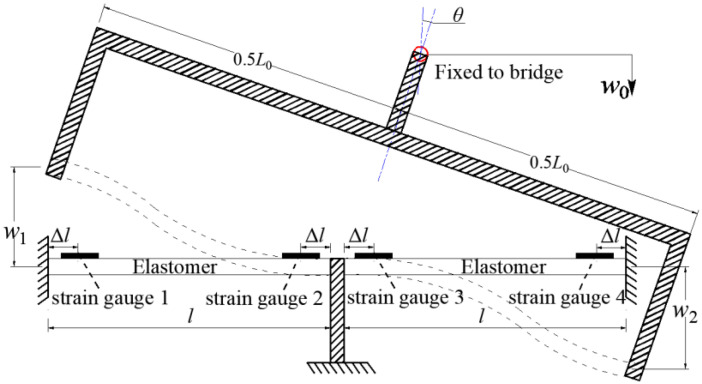
Deformation illustration of the inclinometer under bridge rotation.

Figure 2 illustrates the deformation of the inclinometer under bridge rotation, *w*_1_ and *w*_2_ represent the displacements at two ends of the rigid component caused by the rotation of the beam end (downward displacement is positive and upward is negative); *w*_0_ represents the vertical displacement of the beam end, and it is always small. The following equation represents the displacement relationship shown in Figure 2.

(1) $$w_{1}=w_{0}+\frac{L_{0}}{2}\theta$$

(2) $$w_{2}=w_{0}-\frac{L_{0}}{2}\theta$$

(3) $$w_{1}-w_{2}=L_{0}\theta$$

Displacement of the top rigid body causes the bending deformation of the two elastomers. On left elastomer, the values of strain gauges 1 and 2 caused by *w*_1_ can be expressed as follows:

(4) $$\varepsilon_{1}=-\varepsilon_{2}=\frac{3h(l-2\Delta l)}{l^{3}}w_{1}$$

where *l* and *h* are the length and thickness of the elastomer, respectively; and Δ*l* represents the distance between strain gauges and elastomer ends. Similarly, on the right elastomer:

(5) $$-\varepsilon_{3}=\varepsilon_{4}=\frac{3h(l-2\Delta l)}{l^{3}}w_{2}$$

The relation between the rotation angle and the elastomer strain can then be obtained from (Equations (1)–(5)). The fundamental principle of the inclinometer proposed in this work is to convert the small rotation into the strain of the elastomers. The rotation can thus be obtained by measuring the strains. Compared with the direct rotation or displacement measurement, strain measurement has higher resolution and thus can improve the accuracy of bridge rotation sensing. Moreover, all connections of the inclinometer are rigid to reduce potential errors, which can further enhance the accuracy of rotation measurement results.

## 3. Design and Data Acquisition of the Proposed Inclinometer

### 3.1. Design

**Figure 3:**
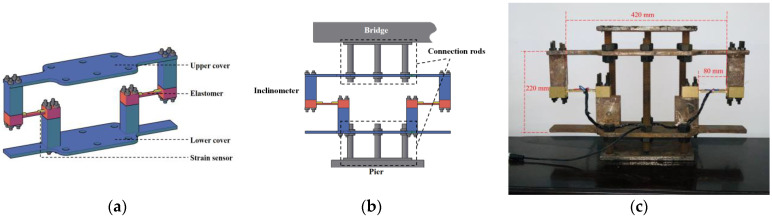
Schematic illustration of the inclinometer: (**a**) device components; (**b**) device installed on bridge; (**c**) prototype of the proposed inclinometer.

Figure 3a,b shows the design of the proposed inclinometer, which consists of upper and lower rigid covers, elastomer, strain gauges, rigid rods, and connecting bolts. The upper and lower covers are designed to be fixed on the superstructure and pier of the bridge, respectively. The elastomer with two strain gauges connects the upper and lower covers. The rigid rods are bolted to the covers for height adjustment on different bridges, which can be disassembled easily and reused. Figure 3c shows the prototype of the proposed inclinometer.

Dimensions and features of each component are summarized in Table 2.

The sensitivity coefficient and measurement range of the proposed inclinometer can be adjusted for different purposes by changing the height, length, and modulus of the elastomer, which is further discussed in the following sections.

### 3.2. Data Acquisition Module

**Figure 4:**
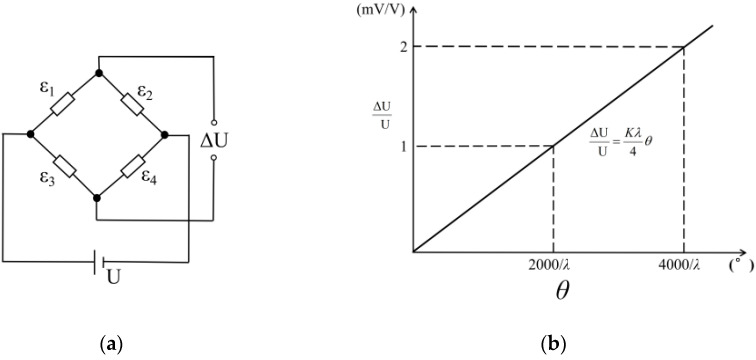
(**a**) Full bridge circuit; (**b**) sensor characteristic curve.

The proposed inclinometer converts the rotation into a strain signal. Thus, based on the current strain gauge data acquisition system, a custom data collection module was designed. As discussed in Section 2, a total of four strain gauges were used on the inclinometer, which can form a Wheatstone full-bridge circuit, as shown in Figure 4a. The conversion relation between the output strain signal *ε* of the full-bridge and rotation *θ* can be established as follows:

(6) $$\varepsilon =\varepsilon_{1}-\varepsilon_{2}+\varepsilon_{3}-\varepsilon_{4}=\frac{6h(l-2\Delta l)L_{0}}{l^{3}}\theta$$

Let

(7) $$\lambda =\frac{6h(l-2\Delta l)L_{0}}{l^{3}}$$

Then

(8) ε=λ⋅θ

It is evident from Equations (6)–(8) that there is a linear relation between the full-bridge output and the rotation. In Figure 4a, U is the input voltage of the strain circuit, ΔU is the output voltage. Based on the characteristics of the strain monitoring circuit, the output voltage and rotation angle satisfy:

(9) $$\frac{\Delta U}{\;U}=\frac{K}{4}\lambda \cdot \theta$$

where *K* is the sensitivity coefficient of the strain gauge and it is usually equal to 2. The inclinometer sensitivity coefficient *λ* is determined by Equation (7). By adjusting the elastomer height *h* and length *l*, strain gauge position Δ*l*, and inclinometer length *L*_0_, the sensitivity coefficient can then be adjusted for different measuring purposes.

The theoretical characteristic curve of the inclinometer is shown in Figure 4b, which indicates the output signal of the inclinometer is linearly correlated to the measured rotation.

**Figure 5:**
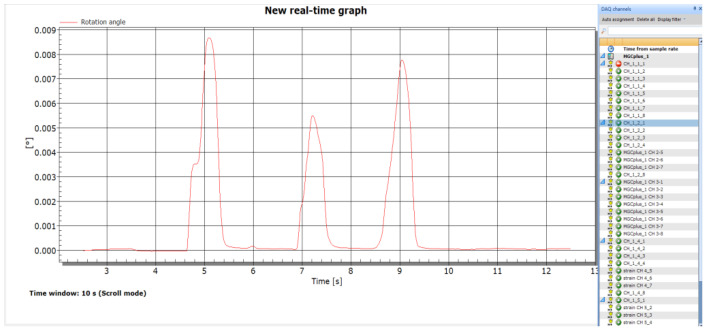
Data acquisition module interface.

As shown in Figure 5, the HBM dynamic testing system (with an MGCplus data acquisition system) and its supporting Catman Easy software were utilized as the data acquisition module of the proposed inclinometer.

The data acquisition module interface contains the real-time output curve of measured rotation versus time (right part in Figure 5) and inclinometer list (left column in Figure 5), which is designed for multiple inclinometers measurement for in-situ application. The maximum data acquisition frequency is determined by the HBM system, which is 9.6 kHz.

### 3.3. Calibration Test

**Figure 6:**
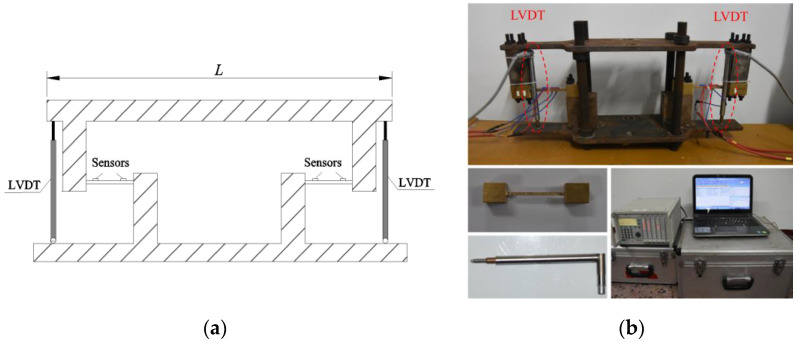
(**a**) Schematic illustration and (**b**) laboratory setup of the calibration test.

For the calibration test, as shown in Figure 6, the lower cover was fixed, while the upper was set to undergo dynamic rotation in the longitudinal direction. Two LVDTs were placed at both ends of the cover to monitor the vertical displacement, from which the actual rotation can be derived. In this study, we used an HBM LVDT displacement transducer with a precision of 0.001 mm. The displacement transducer measurements are provided as *w*_1_ and *w*_2_, and the real-time rotation angle is calculated by (*w*_1_ − *w*_2_)/*L*.

**Figure 7:**
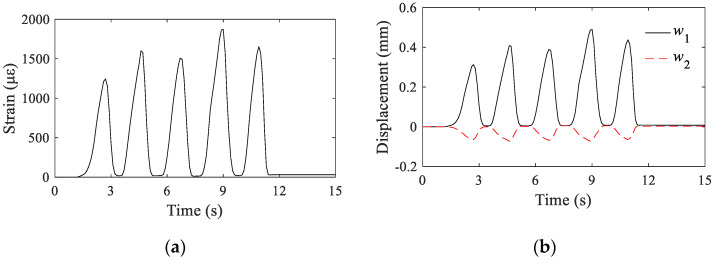
Strain and displacement curves under repeated load (**a**) strain curve; (**b**) displacement curve.

**Figure 8:**
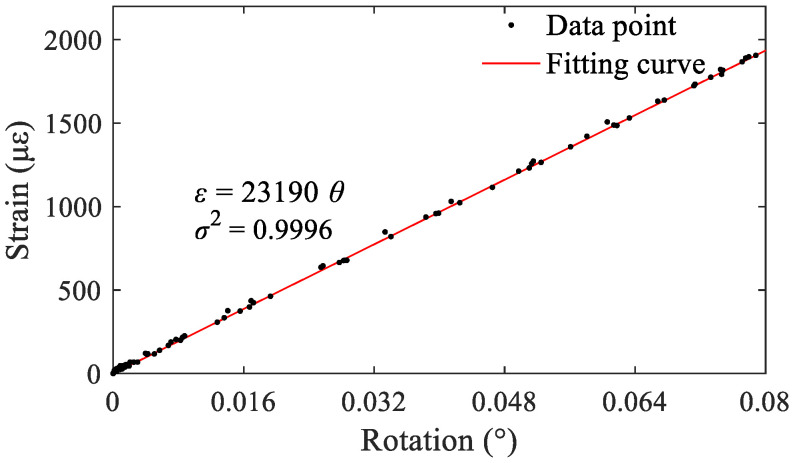
Rotation-strain curve.

The calibration process was conducted by repeatedly applying a weight on the inclinometer and then release it. Sampling rate of 50 Hz was chosen to ensure that the dynamic response of the inclinometer under such circumstance can be adequately captured. The time-history curve of the strain output is shown in Figure 7a. Similarly, time-history curves of the relative displacements measured by the LVDTs are shown in Figure 7b. The corresponding relation between the output strain and input rotation *θ* is shown in Figure 8. It is evident that there is a good linear correlation between the two parameters.

Figure 8 shows that even a small rotation angle input can result in a large strain of the elastomer, which means the proposed inclinometer has ultrahigh sensitivity. After carrying out five calibration tests, the value of the coefficient *λ* was averaged to be 23,190 με/°. Factors such as the physical parameters (elastic modulus) of the elastomer, manufacturing errors, and strain gauge installation errors can cause a discrepancy between the real and theoretical value of *λ*. Nevertheless, the proposed inclinometer can significantly amplify the rotation signal, which proves that it is suitable for high-precision dynamic monitoring of bridge rotation. 

## 4. Inclinometer Characteristic Parameters

### 4.1. Measurement Range

The measurement range of the inclinometer is determined by the elastic modulus and dimensions of the elastomer. The elastomer used in this study was fabricated of brass. This material has a relatively high allowable stress ([*σ*] = 369 MPa) and low elastic modulus (E = 110 GPa), making it easy to deform. Based on the inclinometer design, Δ*l =* 12 mm, *L*_0_ = 420 mm, the calculated range can be obtained by the two steps as follows: 

The allowable strain [*ε*]:

(10) $$\left[\varepsilon \right]=\frac{\left[\sigma \right]\left(l-2\Delta l\right)}{El}=2348\mu \varepsilon$$

The maximum rotation angle:

(11) $$\left[\theta \right]=\frac{2l^{3}}{3h(l-2\Delta l)L_{0}}\left[\varepsilon \right]=0.39{}^{\circ}$$

As shown in Equation (11), the rotation measurement range is determined by elastomer length *l*, height *h*, strain gauge position Δ*l*, and inclinometer length *L_0_*. Therefore, by adjusting the elastomer dimension and inclinometer setup, the maximum measurement range of the proposed inclinometer can be tuned for different purposes. The above calculation is based on the assumption that the centerline of the inclinometer is the same with the rotation axis of the bridge, which means *w*_1_ = −*w*_2_. In real application, this may not be satisfied and thus the range will be slightly decreased. However, the maximum rotation specified in the current bridge design code is 0.115°. Therefore, the range of the inclinometer is sufficient to meet the requirements for beam-end rotation monitoring. In addition, the measurement range can be adjusted by changing the elastomer material, size of the elastomer, or the effective length of the upper cover.

### 4.2. Measurement Accuracy

The precision of an instrument refers to the difference between the measurements and the real values. The inclinometer accuracy relies on the HBM MGC Plus dynamic data acquisition system. According to previous study [43], the relative error of the HBM dynamic monitoring system for strain measurements is 0.1%, and the relative error, *δ*, for the rotation angle is 0.1%. The maximum error, that is, when the measured rotation angle reaches the measurement range of the present monitoring device, △_max_, is provided by the following:

(12) $$\Delta_{\max}=\delta \left[\theta \right]=3.9\times 10^{-4}\;({}^{\circ})$$

Compared with other inclinometers listed in Table 1, accuracy of the proposed inclinometer is obviously higher. It is worth noting that Equation (12) describes the maximum absolute error of the present device, but the precision level is related to the rotation angle measured during the actual monitoring process, which will generally not exceed the monitoring range of the present device. Using actual monitoring data from Section 4.2 as an example, the maximum rotation angle of the beam end during the selected monitoring period was 6.85 × 10^−3^ (°), and the corresponding monitoring error was 6.85 × 10^−6^ (°). This device had obvious advantages in terms of its accuracy and can be used to accurately monitor small rotations of beam ends.

### 4.3. Measurement Resolution

**Figure 9:**
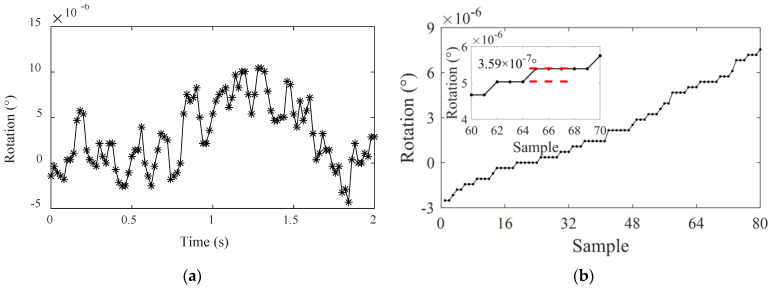
Resolution test chart (**a**) actual test curve; (**b**) resolution.

Resolution of an instrument refers to the ability to capture the smallest change in the measurand. For the proposed inclinometer, it first relies on strain measurement resolution. Then amplification of the rotation angle by the inclinometer also increases the resolution. The actual resolution can be obtained based on the calibration data in Section 3.3. One of the rotation time-history curves was selected, as shown in Figure 9a, and the measured data was sorted based on the magnitude, as shown in Figure 9b.

It is evident from Figure 9b that when the rotation angle values output by the present device are sorted, an obvious stepwise increase can be observed, and the minimum change in the rotation angle, that is, the device resolution, can be determined to be 3.59 × 10^−7^ (°). The difference in the rotation angles between adjacent data points in Figure 7a can be expressed as N × 3.59 × 10^−7^ (N is an integer). It is evident that compared with the parameters of the commonly used inclinometers listed in Table 1, the present device is more advantageous in terms of its resolution. On the other hand, the present rotation monitoring device could not only satisfy the high precision and resolution requirements for bridge rotation monitoring, but its precision and resolution performance were also unaffected by the monitoring frequency.

### 4.4. Linearity and Repeatability

**Figure 10:**
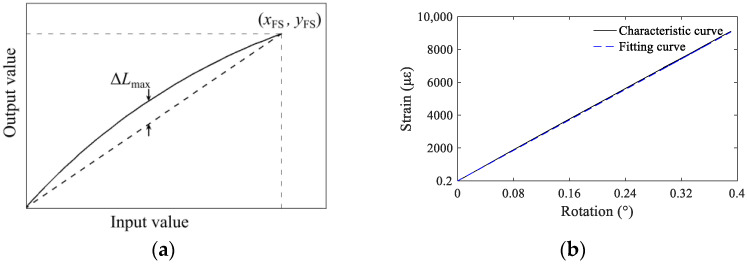
(**a**) Schematic diagram of linearity curve; (**b**) actual linearity of the proposed inclinometer.

Linearity refers to the coincidence degree between the actual characteristic curve of the transducer and the reference line, as shown in Figure 10a. The nonlinear error, *e*_1_, of the characteristic curve can be calculated using Equation (12) [44]. A smaller nonlinear error indicates less the influence of the initial state on the characteristic relationship between the input value and the output value of the transducer. Thus, any point within the measurement range can be used as the initiation point for relative monitoring. The calibration test data in Section 3.3 were used as a basis to determine the actual linearity of the present device. Different rotation angle values were input into the monitoring equipment within its measurement range to obtain the actual characteristic curve of rotation–strain conversion, as shown in Figure 10b. Using the line between the starting point and the maximum point of the measured data as the reference line, the nonlinear error of the present monitoring equipment was calculated to be 0.76%.

(13) $$e_{1}=\frac{\left|\Delta L_{\max}\right|}{y_{\mathrm{FS}}}\times 100\%$$

**Figure 11:**
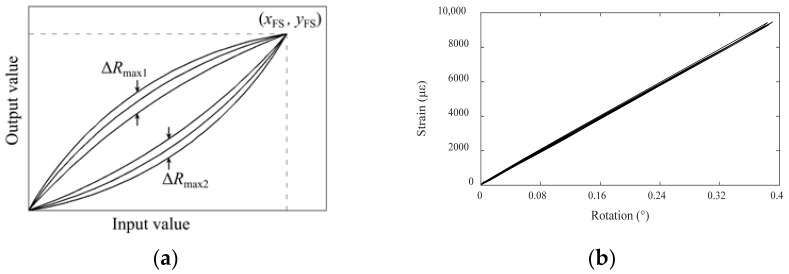
(**a**) Schematic diagram of repeatability characteristics; (**b**) actual repeatability curve of the proposed inclinometer.

Similarly, repeatability describes the degree of non-coincidence for the input–output curve when the transducer input changes repeatedly, as shown in Figure 11a. The repeatability error can be calculated using Equation (14). If the degree of non-coincidence is large, the output values differ greatly for the same input signal, which also affects the monitoring accuracy. Based on the repeatability characteristic curve of the experimental monitoring data, as shown in Figure 11b, the repeatability error was calculated to be 0.82%.

(14) $$e_{2}=\frac{\left|{\Delta R}_{\max}\right|}{y_{\mathrm{FS}}}\times 100\%$$

The linearity and repeatability of a transducer are also related to its manufacturing technology and strain gauge bonding technology. In general, the actual characteristic curve of the proposed device had good linearity and high coincidence, and its nonlinear error and repeatability error were very small, indicating that the device had good linearity and repeatability.

## 5. In-Situ Experiment

### 5.1. Experiment Overview

**Figure 12:**
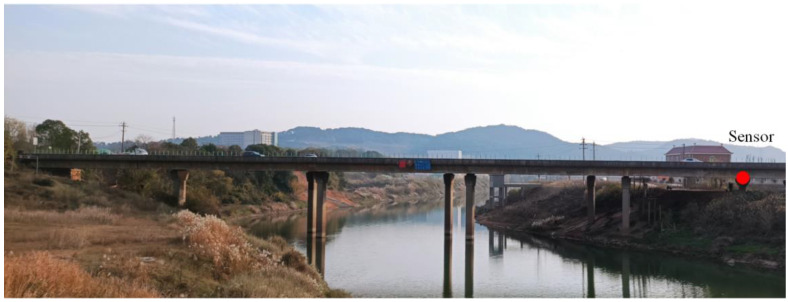
Bridge overview and installation point (red dot) of the inclinometer.

To further verify the feasibility and function of the proposed inclinometer, an expressway bridge called Jinjiang Bridge in Changsha City, Hunan Province (as shown in Figure 12), was selected to conduct in-situ experiments. Real-time beam-end rotation data generated by the random vehicle loads were captured and analyzed. The monitored bridge is a five-span (each span measures 60 m) continuous bridge aligned in the east–west direction. The superstructure is a prestressed concrete single-cell box girder. No lane closures were required during the experiment. Therefore, the beam-end rotation monitoring was carried out during the actual operation of the bridge.

**Figure 13:**
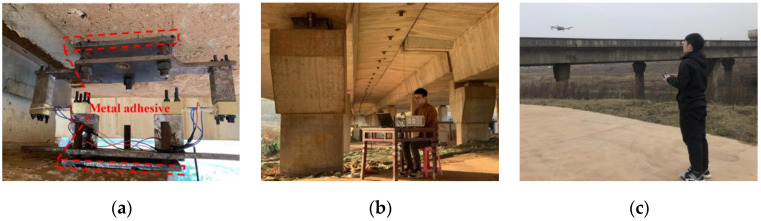
On-site installation of BRT-1 rotation monitoring device (**a**) device installation; (**b**) overall setup; (**c**) UAV photography.

In the field experiment, the inclinometer was set at the beam end at the supporting pier of the first span, as shown in Figure 12. Taking the pier as the fixed reference point, the rotation angle of the beam end relative to the pier was measured in real-time. The gap between the pier and the bridge was 29 cm, which was slightly higher than the height of the inclinometer main part. Thus, the connection bolts were used to adjust height for correct installation. High-strength structural epoxy with maximum tensile strength of 6.5 MPa was used to fix the inclinometer on the beam and pier, as shown in Figure 13a. A temporary working platform was set up near the monitoring point to for data acquisition and the sampling frequency was 50 Hz. The in-situ monitoring setup is shown in Figure 13b. An unmanned aerial vehicle (UAV) was used to photograph the bridge deck to synchronously record the vehicle loads, as shown in Figure 13c.

### 5.2. Results Analysis

**Figure 14:**
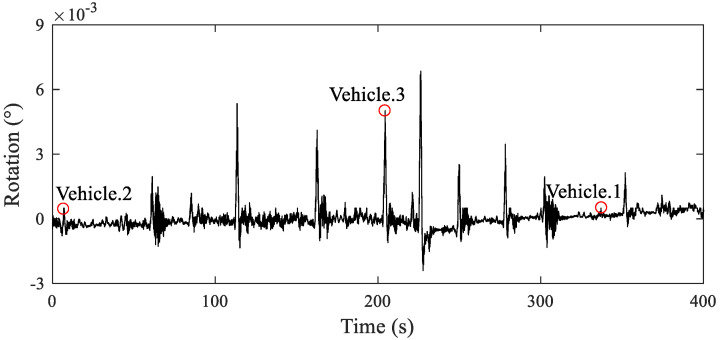
Time-history of typical beam-end rotation response.

During the experiment, the dynamic changes in the beam-end rotation were recorded. The UAV captured video was utilized to mark each passing vehicle with time synchronization to the rotation data. A typical 10 min period of data was selected for analysis, as shown in Figure 14. It was evident that when a vehicle passed the monitored span, rotation response of the beam end was effectively captured by the proposed inclinometer. The captured data were compared with the UAV photos and it showed that different amplitude signals correlated to different vehicles sizes (small, medium, and large). 

**Figure 15:**
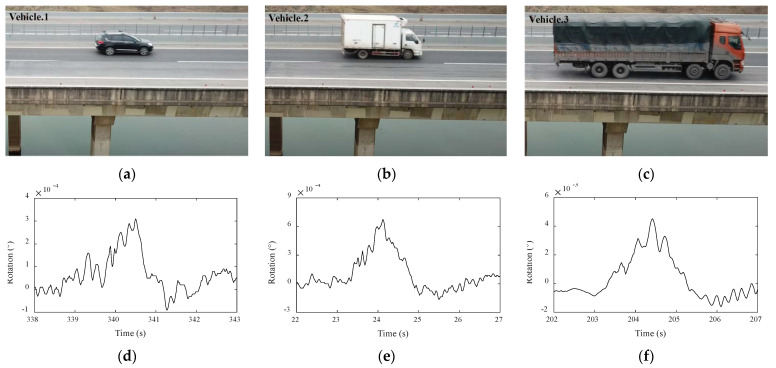
Beam-end rotation monitoring data under different vehicle loads (**a**) small vehicle; (**b**) medium vehicle; (**c**) large vehicle; rotation response to (**d**) small vehicle; (**e**) medium vehicle; (**f**) large vehicle.

The time-history data in Figure 14 were divided into segments to isolate each rotation response when only a single vehicle crossed the span. Three typical data for different sizes of vehicles, together with the corresponding photos captured by the UAV, are shown in Figure 15. Figure 15a–c show the different sizes vehicles in each section, and Figure 15d–f show the corresponding beam-end rotation response curves. It is evident that the time required by each vehicle to pass the monitored span was approximately 2 s, indicating that the three vehicles traveled across the bridge at a high speed of approximately 110 km/h (speed limit of the bridge is 120 km/h). The changes in the amplitude of the rotation angle caused by vehicle 1, vehicle 2, and vehicle 3 were 6.2 × 10^−4^, 3.2 × 10^−4^, and 46.8 × 10^−4^ (°), respectively. The beam-end rotation and corresponding vehicle sizes and weight are listed in Table 3.

The monitoring point was located at the fixed support near the expansion joint close to the end of the bridge. Therefore, when a vehicle passed over the expansion joint, the rotation data inevitably contained some vibration interference. Nevertheless, the proposed device was still able to accurately capture the rotation response of the beam end throughout the entire experiment. When a 35-ton vehicle passed the bridge, the magnitude of the beam-end rotation response was approximately 4% of the specified limit (0.115°) for highway bridges. The response curve clearly reflected the entire process and contained very little noise. Furthermore, even when smaller vehicles of 2–5 tons crossed the bridge at a high speed, the beam-end response was also clearly captured by the proposed inclinometer. The above experimental results showed the feasibility and advantages of the proposed inclinometer in terms of resolution and precision. Therefore, it can be used effectively to capture the rotation responses of in-service bridges. 

## 6. Conclusions

In this study, we developed a new elastomer-based inclinometer for bridge rotation monitoring. The monitoring mechanism of the proposed device involved converting a small displacement generated by the rotation of a rigid component into the strain of an elastomer, and then using strain gauges to obtain a highly accurate beam-end rotation signal. Compared with other methods, the proposed inclinometer can capture the small rotation more accurately.

Based on theoretical analysis and calibration test results, measurement range, accuracy, resolution, linearity, and robustness were all analyzed. The results showed that the measurement range of the proposed inclinometer met the current bridge design code; the relative precision was 1/1000, the maximum absolute error was 3.19 × 10^−4^ (°), the resolution was 1.365 × 10^−6^ (°), the nonlinear error and repeatability error were low, and its performance parameters were better than most commercial rotation transducers. 

The proposed inclinometer was tested on an in-service bridge. The device was installed between the pier and the bridge superstructure to accurately capture the rotation response under the actions of moving vehicles. The case study results showed that the inclinometer output curve clearly reflected the beam-end rotation even for small vehicles crossing the bridge at a high speed. The proposed inclinometer has obvious advantages in terms of its resolution and precision, and can thus be used effectively to monitor the rotation responses of bridges in the future.

## Figures and Tables

**Table 1:** Technical specifications of commercially available inclinometers.

Model	Country of Origin	Measurement Range (°)	Resolution in Degrees (°)	Precision in Degrees (°)	Sampling Rate (Hz)
DNS	Germany	±85	3 × 10^−3^	±3 × 10^−2^	100
JDI 100	USA	±1	1 × 10^−4^	±4 × 10^−3^	125
JN2101	Germany	±45	1 × 10^−3^	±1 × 10^−2^	20
ACA2200	Japan	±0.5	1 × 10^−4^	±3 × 10^−3^	20
ZERO-TRONIC	Switzerland	±0.5	1 × 10^−4^	±3.5 × 10^−4^	10
T935	UK	±1	6 × 10^−5^	±4 × 10^−4^	10

**Table 2:** Dimensions and features of the inclinometer components.

No.	Component	Size/(mm)	Feature
1	Upper cover	420 × 150 × 8	Convert rotation to strain
2	Lower cover	420 × 150 × 8	Fix reference plane
3	Elastomer	80 × 10 × 5	Elastic sensitive element
4	Limit rod	Φ20	Limit before test
5	Connection rods	100 × 100 × 8	Fix the device to the bridge
6	Strain gauge	5 × 3	Sensing elastic beam strain signal

**Table 3:** Rotation responses generated by different types of vehicles.

Vehicle Size	Small	Medium	Large
Approx. vehicle weight (ton)	2	4	35
Rotation response (×10^−4^ (°))	3.2	6.2	46.8
